# Steroid efficacy in Meralgia Paresthetica: A systematic review and meta-analysis

**DOI:** 10.12669/pjms.40.1.8162

**Published:** 2024

**Authors:** Wajid Jawaid, Samna Haider, Navaira Shoaib, Naveen Azhar, Arooma Shaukat Siddiqui

**Affiliations:** 1 Wajid Jawaid, FCPS Neurology. Department of Neurology, Dow University of Health, Sciences & Dr. Ruth K. M Pfau Civil Hospital, Karachi, Pakistan. Karachi, Pakistan; 2 Samna Haider, MBBS. Department of Internal Medicine, Dow University of Health Sciences, Karachi, Pakistan; 3 Navaira Shoaib, MBBS. Department of Internal Medicine, Dow University of Health Sciences, Karachi, Pakistan; 4 Naveen Azhar, MBBS. Department of Internal Medicine, Dow University of Health Sciences, Karachi, Pakistan; 5 Arooma Shaukat Siddiqui, MBBS. Department of Internal Medicine, Dow University of Health Sciences, Karachi, Pakistan

**Keywords:** Meralgia paresthetica, Steroids, Pain management, Lateral femoral cutaneous neuropathy, Entrapment neuropathy

## Abstract

**Objective:**

To determine the efficacy of steroid injections for pain relief in patients with meralgia paresthetica (MP).

**Methods:**

All the literature published until March 2023 was explored from several databases, including EBSCO, PubMed, EMBASE, Cochrane Library, Google Scholar, and Scopus. Articles investigating the change in pain status of MP patients after steroid injection were included. The primary outcomes were complete pain relief, pain scores at 15 days and one month after intervention. When compared to the baseline, the secondary outcomes for the steroid group included pain scores at the end of treatment and quality of life, which were further evaluated by two factors, namely mental and physical health.

**Results:**

The analysis of the studies validated that steroids were significantly successful in providing complete pain relief (p-value = 0.00001), and in reducing the pain score of patients with meralgia paresthetica at 15 days (p-value = 0.02), but not at one month (p-value = 0.79) as compared to the control group. The analysis did not reveal any significant subgroup differences among various steroids (P = 0.52; CI: 0.01 - 0.10; RR: 0.04; I^2^ = 0%). Mental health (MD = 4.23; 95% CI = 0.42 to 8.03; p = 0.03, I2 = 0%) was significantly improved in the steroid group when compared with baseline.

**Conclusion:**

Steroids injections can play an important role in improving symptoms and complications of meralgia paresthetica, especially in the short term.

## INTRODUCTION

A neurological ailment called Meralgia Paresthetica (MP), also known as Bernhardt-Roth Syndrome, causes numbness, pain, burning, and tingling sensations in the anterolateral region of the thigh.^1^ This disorder results from the compression of the lateral femoral cutaneous nerve (LFCN).^2^ LFCN is a branch of the lumbar plexus that emerges from posterior divisions of the anterior rami of L2 and L3 spinal nerves.^3^ The LFCN crosses the iliacus muscle after emerging from the lateral border of the psoas major and heads toward the anterior superior iliac spine (ASIS) before passing beneath the inguinal ligament, where there is high chance of entrapment.^3^,^4^ Several factors, like tight clothing, obesity, pregnancy, seat belts, diabetes, etc., can contribute to the compression of LFCN.^5^

MP can affect all ages, with incidence of 4.3 cases per 10,000 person years with predomination of adult males. Its incidence in diabetics is around 247 cases per 100,000 person years. Out of 10,000 deliveries, Postpartum LFCN compression is reported in around one to 58 cases.^6^ Several studies have concluded its incidence in patients who underwent spinal surgeries and in intensive care unit patients after prone position ventilation.^7^,^8^ Furthermore, it is also seen as a late complication in COVID-19 patients.^9^ More data has been published depicting its prevalence among athletes, gymnasts, body builders and various individuals doing strenuous exercise.^6^ It produces signs and symptoms associated with other ailments, for example lumbar spine pathology, making the diagnosis challenging. The years lost to premature mortality would not be attributable to MP because it is not a life-threatening condition. Nonetheless, depending on how severe it is and how well it responds to therapy, it can cause impairment. It can influence everyday activities and quality of life for those who have MP to feel pain, numbness, tingling, or discomfort in the affected area. Patients may develop antalgic gait. However, it’s important to remember that MP is frequently treatable and doesn’t usually result in severe impairment.^6^ Severe pain can be debilitating, and the patient often seeks relief from a conservative therapeutic approach involving analgesics and alternative pharmacological options.^5^ Other treatment strategies include removing aggravating factors, steroid injection, neurolysis-mediated decompression, and surgical neurectomy. However, further studies, analyses, and trials are needed to conclude a definitive treatment plan. In this study, we aimed to review and analyze the established literature to evaluate the effectiveness of corticosteroid injection as a therapeutic measure for MP.

## METHODS

### Search Strategy

Our search strategy was designed using the PICO (Population, Intervention, Comparison and Outcome) query format as: “In individuals with meralgia paresthetica (Population) who had steroid injection (Intervention), how did different steroid injections (Comparison) affect patient-reported outcomes of complete pain relief, pain scores and quality of life (Outcome)?”

The Preferred Reporting Items for Systematic Reviews and Meta-analysis (PRISMA) declaration and the Cochrane Handbook for Systematic Reviews of Interventions and Meta-analysis were used to direct this meta-analysis.^10^,^11^

### Criteria for searching and selecting literature

From the inception to March 2023, we extensively scanned many databases, including EBSCO, PubMed, EMBASE, Cochrane Library, Google Scholar, and Scopus, with the following keywords: “lateral femoral cutaneous neuropathy” or “meralgia paresthetica“ or “lateral femoral cutaneous nerve entrapment”; “steroid” or “injection”; “pain” or “pain management”; and “ultrasound”. We searched every article in the reference list of the included studies for the meta-analysis to ensure more qualifying results.

### Inclusion and exclusion criteria

The following were the inclusion criteria:

- Randomized and non-randomized studies were used as the study design.
- Patients that have been diagnosed with meralgia paresthetica.
- Steroid injection was used as an induction therapy.

Conversely, studies that were not published in English, case reports, editorials, letters, comments, and conference abstracts, studies that involved animals or healthy individuals, and studies that did not primarily employ steroid injection as the intervention were excluded from the exploration.

### Data handling and outcome assessment

The first author, the overall patient count, age, the severity of the pain, the duration of the disease, and the intervention strategies made up the baseline data that were taken from the original research. Two of the researchers gathered the data separately, disagreements were resolved concurrently and, where necessary, with the assistance of a third reviewer. The data was presented graphically, and WebPlotDigitizer software version 4.2 was used to estimate means and standard deviations from the graph.^12^

The primary outcomes were complete pain relief and a reduction in pain levels at 15 days and one month. The pain outcome i.e., complete pain relief, was further quantified in two tiers: the comparison of various steroids and whether the steroid injection administration was ultrasound-guided or not. Reductions in pain levels were measured at 15 days and one month after intervention in both the steroid and control groups. The secondary outcomes were pain scores, freedom from neuropathic medications and quality of life further assessed by two variables, i.e., mental health and physical health, in the steroid group following intervention when compared with baseline.

### Assessment of the quality and bias risk

The Cochrane Collaboration’s tool for analyzing the risk of bias was used to classify the quality of the randomized studies as poor, moderate, or high.^13^ The NIH Quality Assessment Tool for Observational Cohort and Cross-Sectional Studies was used to evaluate the non-randomized studies; the studies were further rated as good, fair, or poor quality.^14^,^15^ The research group got together to work out any disagreements and establish concordance.

### Statistical analysis of the included studies

The risk ratios for the dichotomous outcome measures were calculated using the Mantel-Haenszel method. The inverse variance tool was applied to determine mean differences (MD) for continuous data sets. All estimates were given a 95% confidence interval (CI). When evaluating heterogeneity, the Higgins I^2^ statistic was utilized, and a value of I^2^> 75% denoted significant heterogeneity.^16^ The meta-analyses were conducted using the random-effects model. Furthermore, we executed a subgroup analysis centered on the steroid drug class, as well as the use of ultrasonography and a blind approach. A p-value less than 0.05 indicated that the results were statistically significant. Review Manager Version 5.4 of the Cochrane Collaboration tool was used to do the entire data analysis.^17^

## RESULTS

### Approach for searching the literature

**Fig.1:**
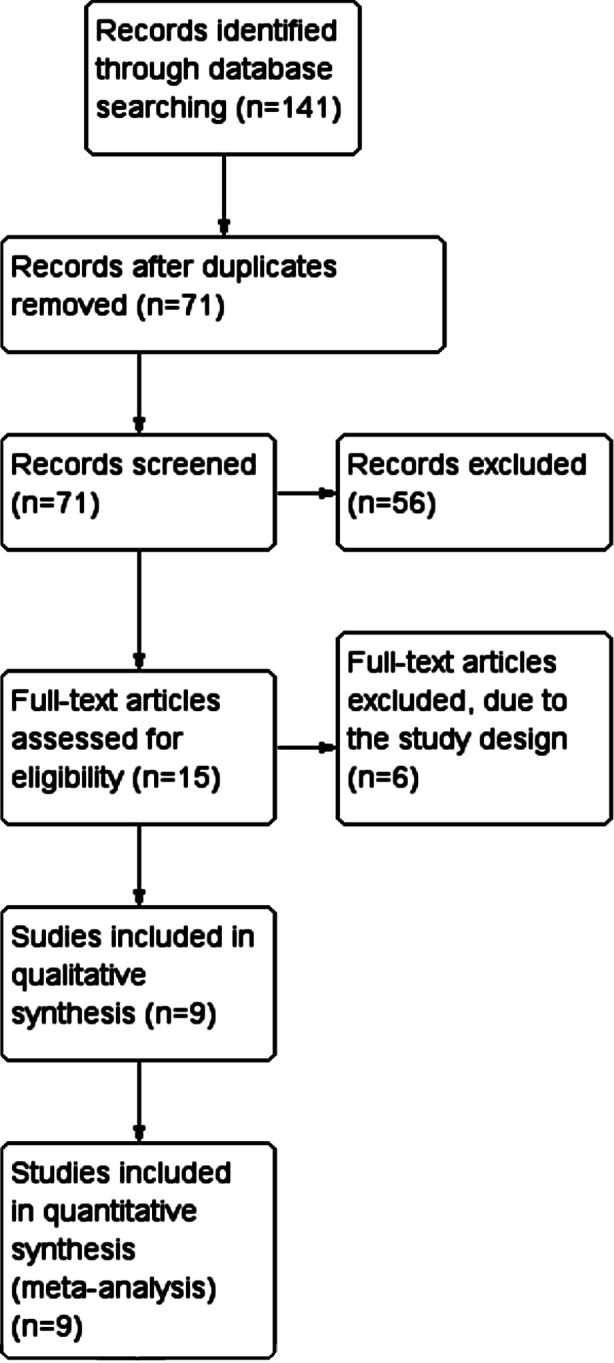
Flow diagram of the search strategy.

Indicates the strategy of search in accordance with the guidelines of PRISMA (Fig.1). Nine studies met our inclusion criteria, of which two were randomized trials,^18^,^19^ while the other seven publications were non-randomized studies.^20^-^26^

### Characteristics of the Study and Quality Evaluation

The baseline attributes of the publications used during the analysis (Table-I). The cumulative sample size for these studies was 243. Using the Cochrane Collaboration’s tool for measuring the risk of bias, the RCTs were of high quality. Both RCTs were found to have a low risk of bias. The NIH Quality Assessment Tool gave four non-randomized studies a good quality rating and three non-randomized studies a fair quality rating.

**Table-I:** Baseline attributes of included studies..

Study ID	Country	Number of patients	Age in years Mean ± sd (range)	Bilateral cases	Number of females	VAS score at baseline	Mean pain duration years (range)	Steroid name	Steroid dose	Concomitant drug	Nerve stimulator guided	Ultrasound guided	Mean number of sessions	Mean follow up duration months (range)
Kilic 2020	Turkey	17	51.23±12.58	-	6	1.88 ± 3.06	1.063 ± 1.16	Betamethasone	5 mg	2% prilocaine (2ml)	No	Yes (7-13 MHz)	1	1
Kloosterziel 2017	Netherlands	10	56.6 ± 8.9	-	4	7.4 ± 1.8	0.958 (median)	Methylprednisolone	80 mg	lidocaine (20 mg)	Yes (1 Hz, <10 mA)	No	1	3
Ahmed A 2016	India	6	46 ± 10	-	4	8.83 ± 0.75	0.4 (0.3-0.5)	Methylprednisolone	10 mg	0.25% Bupivacaine (4 ml)	Yes. (50 Hz, <0.5 mV)	Yes (6-13 Hz)	2	3
Elavarasi A 2019	India	8	51	1	4	-	-	Triamcinolone acetonide	40 mg	-	No	No	2.5	25 (4-41)
Haim A 2006	Israel	79	43 (16-73)	2	59	-	2.83 (1.08-4.66)	Betamethasone	7 mg	-	No	No	2.03	39.6 (12-156)
Ivins GK 2000	United States of America	14	11 (30-65)	1	9	-	-	Methylprednisolone	-	Bupivacaine	No	No	2.64	49 (38-60)
Klauser AS 2015	Austria	20	61 (46-75)	-	11	8.45 ± 1.66	-	Triamcinolone acetonide	10 mg	0.5% Bupivacane (5 ml)	No	Yes	2.25	12
Okur SC 2017	Turkey	38	55.16 ± 9.99	-	23	7.79 ± 1.08	0.53	Triamcinolone acetonide	20 mg	Prilocaine (4 ml)	No	Yes (5-13 MHz)	1	1
Tagliafico A 2011	Italy	20	39 (23-66)	1	13	8.1 ± 2.1	-	Methylprednisolone	40 mg	2% Mepivacaine (8 ml)	No	Yes (7-17 MHz)	1.2	3

### Primary outcomes: complete pain relief, pain scores at 15 days, and at one month

**Fig.2:**
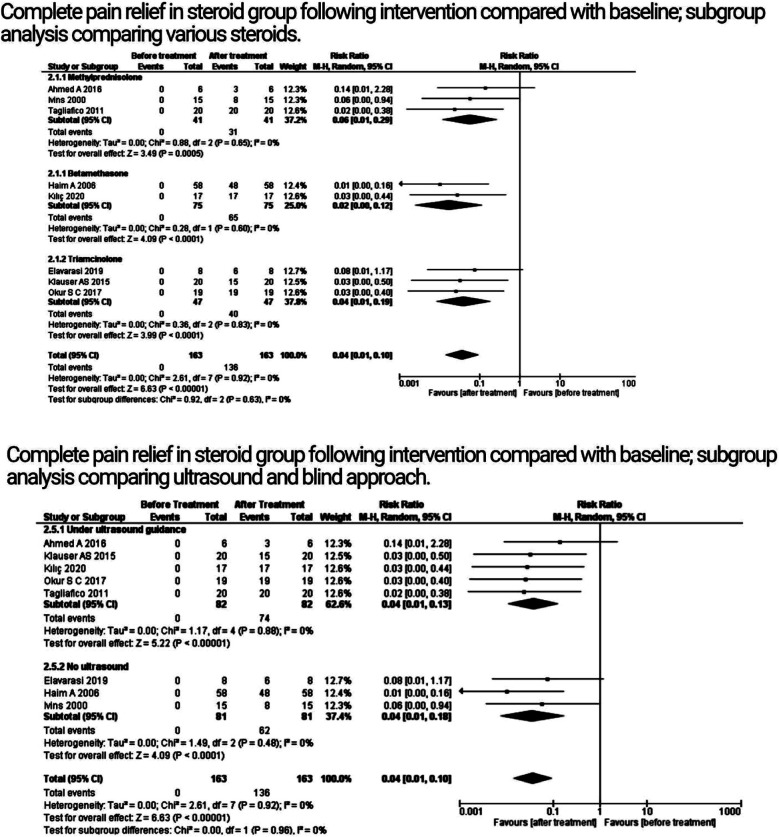
Analysis of primary outcomes; subgroup analysis.

**Fig.3:**
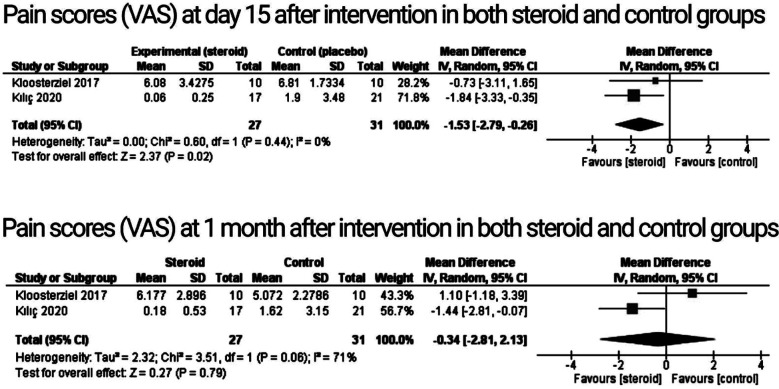
Analysis of primary outcomes; pain scores.

The primary outcomes were analyzed using the random-effects model (Fig.2 and Fig.3). Steroids had a significant impact on pain relief when compared to baseline, according to the pooled analysis of eight studies that reported the outcome of complete pain relief (P < 0.00001; CI: 0.01 - 0.10; RR: 0.04, I^2^ = 0%). The administration of various glucocorticoids (e.g., triamcinolone, methylprednisolone, and betamethasone) varied between investigations. As a result, we used subgroup analyses to stratify the meta-analysis. For the purpose of providing complete pain relief, a subgroup analysis was conducted comparing the administration of different steroids: betamethasone (P < 0.0001; CI: 0.00 - 0.12; RR: 0.02; I^2^ = 0%), triamcinolone (P = 0.0002; CI: 0.01 - 0.09; RR: 0.04; I^2^ = 0%), and methylprednisolone (P < 0.0001; CI: 0.02 - 0.25; RR: 0.06; I^2^ = 0%). The analysis did not reveal any significant subgroup differences among various steroids (P = 0.52; CI: 0.01 - 0.10; RR: 0.04; I^2^ = 0%).

Each experiment had a different method for administering steroids, whether in the presence or absence of ultrasound control. As a result, we used subgroup analyses to categorize the meta-analysis, which contrasted the utilization of ultrasonography (P < 0.00001; CI: 0.01 - 0.13; RR: 0.04; I^2^ = 0%) versus the absence of ultrasound (P < 0.0001; CI: 0.01 - 0.18; RR: 0.04; I^2^ = 0%) with the aim to facilitate overall pain relief. The analysis failed to find any discernible subgroup differences between the two approaches (P: 0.96; CI: 0.01 - 0.1; RR: 0.04; I^2^ = 0%).

Both randomized controlled trials reported pain scores at 15 days and at one month, as determined by the visual analogue scale (VAS). The findings revealed that steroid injection therapy would significantly reduce the pain score in meralgia patients as contrasted to the control arm at day 15 (p = 0.02; CI: -2.79 to -0.26; MD: -1.53), without any heterogeneity in the RCTs (I^2^ = 0%; heterogeneity P = 0.44), but with no significant reduction in pain scores at one month (P: 0.79; CI:- 2.81 to 2.13; MD: -0.34; I^2^ = 71%). Because there were only two studies included in the evaluation of this outcome, we were unable to determine heterogeneity using the sensitivity analysis.

### Secondary Outcomes

**Fig.4:**
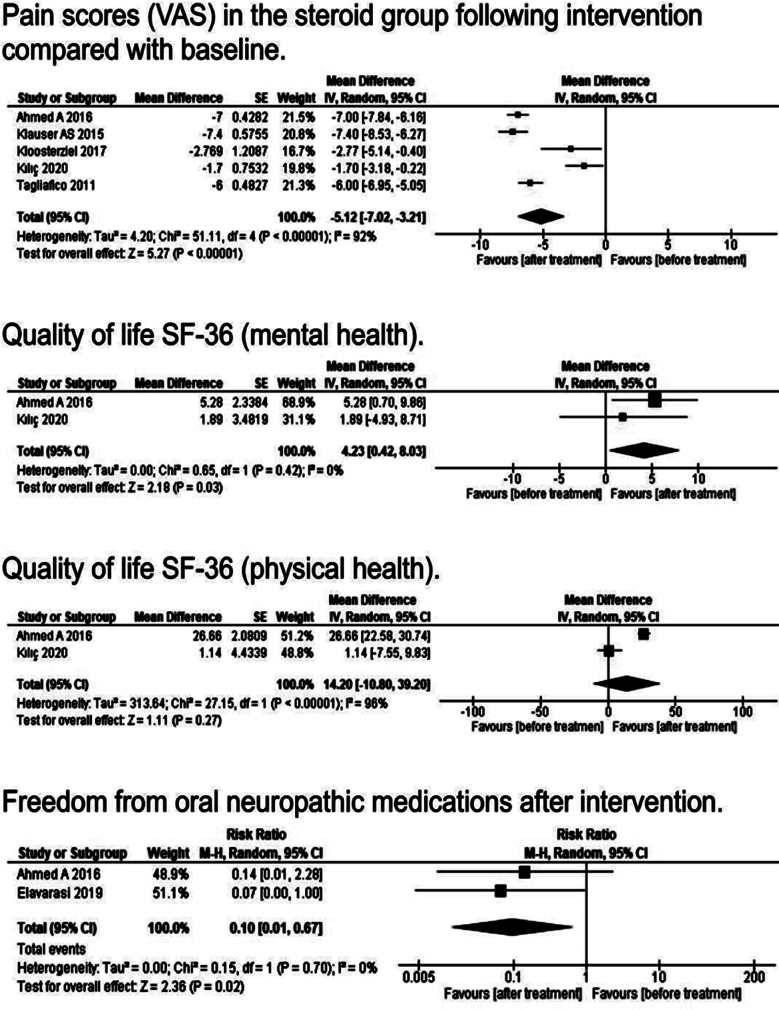
Analysis of secondary outcomes.

The secondary outcomes were also analyzed using random effects models (Fig.4). Nine studies were included to analyze pain scores in the steroid group following steroid injections compared with baseline. Pain scores were reduced significantly when compared with baseline (P: < 0.00001; CI: -7.02 to -3.21; MD: -5.12; I^2^ = 92%). In the quality-of-life outcome, two more aspects were examined: mental health and physical health. The mental health of patients was significantly improved after treatment with steroids (P: 0.03; CI: 0.42 - 8.03; MD: 4.23; I^2^ = 0%). The analysis found no discernible difference between baseline and post-treatment for the physical health result (P: 0.27; CI: -10.8 to 39.2; MD: 14.2; I^2^ = 96%). Two observational investigations examined the connections between steroid injection and variables like freedom from neuropathic medications, negative drug effects, and the cost of treatment in patients with MP. The pooled analysis revealed a remarkable decrease in the intake of prescribed neuropathic medications (P: 0.02; CI: 0.01 - 0.67; RR: 0.1; I^2^ = 0%).

## DISCUSSION

MP is the most widely known nerve entrapment of the lower limbs. The symptoms of MP are similar to L2 or L3 radiculopathy. Consequently, it is often diagnosed late or not at all.^27^ Four years, the treatment of MP has been a point of debate and investigation. Therefore, numerous observational studies and RCTs have been carried out to examine the outcomes of different medications and procedures for pain reduction in MP. Surgical options for treatment are transection and decompression of the LFCN. A systematic review of observational investigations conducted by Payne et al. concluded that not enough data was available to infer the superiority of any of the surgical options over one another.^28^

In the largest meta-analysis of MP treatment using neurectomy, decompression, and injection, we observe that complete pain alleviation following neurectomy occurred most frequently and was accompanied by the least amount of revision surgery. However, neurectomy resulted in loss of sensation, which can be bothersome for some patients, and hence patient preference is very important in the decision of a treatment option. The authors also made the implication that we should keep in mind that no therapy has, as of yet, consistently demonstrated its ability to completely relieve pain.^29^ Our investigation, in contrast to this meta-analysis, focused on the use of various injectable steroids in the management of MP. In addition, we included recent trials not used by the authors of the above meta-analysis.^18^,^19^

Steroids are extensively being used in a wide range of pathologic conditions. Their role in providing pain relief came out as a great development in the field of medicine.^30^,^31^ Tagliafico et al. treated 20 consecutive patients with methylprednisolone acetate and mepivacaine injections under ultrasound-guidance for the treatment of Meralgia paresthetica. Two months following the injection, all patients’ MP symptoms disappeared.^26^ Klauser AS et al., in their clinical trial, administered US-guided corticosteroids at various locations with a follow-up of 12 months. Generally, symptoms completely resolved in 15 patients out of the total 20 and partially resolved in the remaining five.^24^

Triamcinolone acetonide, dexamethasone, and methylprednisolone acetate are the three corticosteroids that are most frequently used to treat MP. We evaluated these steroids’ effectiveness. The current review represents the only meta-analysis comparing various steroids for meralgia paresthetica that offers support for doctors treating patients in selecting the best medication. Furthermore, the researchers of the previous study did not make comparisons among the steroid drug class.^29^,^32^ Our meta-analysis’s findings conclusively show that MP can be treated with any of the three corticosteroids.

The analgesic effect of steroids is related to its anti-inflammatory properties. Steroids stabilize the membrane by inhibiting myelinated C fiber transmission and preventing ectopic release. Local anesthetics selectively block A-delta and C fibers, as well as sodium channels in vasoconstrictor sympathetic nerves, causing nitrous oxide (NO) to be released. NO improves microcirculation in the blood vessels and decreases inflammation. Local anesthetics and glucocorticoids together have been used in MP studies, since this combination extends the time of analgesia.^19^ However, the role of steroids in the treatment of meralgia paresthetica still needs to be clearly defined.

The current research represents the sole meta-analysis that compares the ultrasound guided steroid injection with the blind approach of administering steroids for treating meralgia paresthetica on a single platform. The most recent meta-analysis on this subject included only three studies of ultrasound-guided steroid injection, with no studies on the blind approach included.^32^ Our meta-analysis’s findings clearly demonstrate that MP can be treated effectively both in the absence and presence of ultrasound. The procedure’s effectiveness even in the absence of ultrasound makes it extremely feasible in resource-constrained setups.

The most suitable approach of treating MP may be ultrasound-guided steroid administration, which avoids any possible side effects from systemic medication delivery. While performing the LFCN block, the femoral nerve can be unintentionally blocked, as previously reported in the literature; thus, the utilization of ultrasound enables us to see the dispersion of the drug throughout the block in real time.^33^ In the Klauser study, steroids were administered at various locations under ultrasound guidance, and it was discovered that 14 patients had multiple levels of LFCN involvement. Injections of corticosteroids or local anesthetic agents can be precisely directed to the distended portions of the LFCN using US, which appropriately identifies these regions.^24^ Many of the blind regional nerve blocks fail, which could be explained by the LFCN’s unpredictable relation to no discernible anatomical landmark.^34^

The Lee study utilized a blind injection approach for MP patients thus, requiring numerous additional injections for successful outcomes.^35^ The Ng study evaluated the precision of US in localizing the LFCN in 10 volunteers and 20 cadavers utilizing transdermal nerve stimulators. While effectiveness when using US was 80 percent in volunteers and 84 percent in cadavers, validity when using anatomical landmarks was zero percent in volunteers and five percent in cadavers.^36^ Our study’s findings imply that a precise determination of the degree of nerve alteration and careful administration of the drug in the presence of US supervision may enable the possibility of lowering steroid dosage.

As there are no set dosage recommendations, the range of one to five sessions is common in the practice of steroid injection therapy. There is a subcategory of individuals who report no lingering pain after a single steroid injection over a period of years. Positive outcomes for even the lowest therapeutic dose point to the need for additional research on the ideal intensity and frequency of intervention.

### Limitations of the study

Few restrictions apply to our study, which is typical of all meta-analyses of aggregated data. To begin with, our meta-analysis is based on only two randomized control trials since not many trials are done in this aspect. However, meta-analyses and systematic reviews seek to pinpoint the information gaps and lack of trials in a field. To further explore this issue, more RCTs with larger sample sizes and with a long-term follow-up should be performed in the future. In addition to this, ongoing trials and unpublished data can potentiate the risk of bias.

## CONCLUSION

Meralgia paresthetica presents with severe pain that causes extreme discomfort to the patients. The data analyzed in this study show that steroids injections provide significant relief of pain in the patients of MP, especially in short term, and must be considered as a treatment option. We urge that additional trials in this area be conducted to better determine the utility of this treatment modality in meralgia paresthetica.

### Authors’ contribution:

**WJ:** Conception and design, data analysis and interpretation, drafting and critical revision of the manuscript, responsible and accountable for accuracy and integrity of the work.

**SH:** Conception and design, data acquisition and analysis, drafting and critical revision of the manuscript.

**NS:** Data acquisition and analysis, drafting the manuscript.

**NA:** Data analysis, drafting the manuscript.

**ASS:** Data interpretation, drafting the manuscript.
